# Interleaving cerebral CT perfusion with neck CT angiography. Part II: clinical implementation and image quality

**DOI:** 10.1007/s00330-016-4592-z

**Published:** 2016-09-21

**Authors:** Marcel T. H. Oei, Frederick J. A. Meijer, Willem-Jan van der Woude, Ewoud J. Smit, Bram van Ginneken, Rashindra Manniesing, Mathias Prokop

**Affiliations:** Department of Radiology and Nuclear Medicine, Radboud University Medical Centre, P.O. Box 9101, 6500 HB Nijmegen, The Netherlands

**Keywords:** Multidetector computed tomography, Angiography, Perfusion, Brain, Stroke

## Abstract

**Objectives:**

Feasibility evaluation of the One-Step Stroke Protocol, which is an interleaved cerebral computed tomography perfusion (CTP) and neck volumetric computed tomography angiography (vCTA) scanning technique using wide-detector computed tomography, and to assess the image quality of vCTA.

**Methods:**

Twenty patients with suspicion of acute ischaemic stroke were prospectively scanned and evaluated with a head and neck CTA and with the One-Step Stroke Protocol. Arterial enhancement and contrast-to-noise ratio (CNR) in the carotid arteries was assessed. Three observers scored artefacts and image quality of the cervical arteries. The total z-coverage was evaluated.

**Results:**

Mean enhancement in the carotid bifurcation was rated higher in the vCTA (595 ± 164 HU) than CTA (441 ± 117 HU). CNR was rated higher in vCTA. Image quality scores showed no significant difference in the region of the carotid bifurcation between vCTA and CTA. Lower neck image quality scores were slightly lower for vCTA due to artefacts, although not rated as diagnostically relevant. In ten patients, the origin of the left common carotid artery was missed by 1.6 ± 0.8 cm. Mean patient height was 1.8 ± 0.09 m. Carotid bifurcation and origin of vertebral arteries were covered in all patients.

**Conclusions:**

The One-Step Stroke Protocol is feasible with good diagnostic image quality of vCTA, although full z-coverage is limited in tall patients.

***Key Points*:**

• *Interleaving cerebral CTP with neck CTA (One-Step Stroke Protocol) is feasible*

• *Diagnostic quality of One-Step Stroke Protocol neck CTA is similar to conventional CTA*

• *One-Step Stroke Protocol neck CTA suffers from streak artefacts in the lower neck*

• *A limitation of One-Step Stroke Protocol CTA is lack of coverage in tall patients*

• *Precise planning of One-Step Stroke Protocol neck CTA is necessary in tall patients*

**Electronic supplementary material:**

The online version of this article (doi:10.1007/s00330-016-4592-z) contains supplementary material, which is available to authorized users.

## Introduction

Stroke is one of the leading causes of mortality worldwide [1]. With “time is brain” as the motto, rapid identification of the presence and extent of cerebral ischaemia is essential for treatment decisions. Computed tomography (CT) remains a frequently used modality for patients presenting with symptoms of acute ischaemic stroke to the emergency department because CT is readily and widely available. A CT protocol in the diagnostic work-up of ischaemic stroke usually includes a non-contrast-enhanced CT (NCCT), head and neck CT angiography (CTA) and cerebral CT perfusion (CTP). The combination of CTA and CTP has been shown to be helpful in selecting patients for endovascular treatment [2].

A cerebral CTP acquisition not only contains information about tissue perfusion but also allows for reconstructing a dynamic CTA (4D-CTA) to evaluate the intracranial vessels within the covered region. Several studies have shown that 4D-CTA can replace conventional CTA for the evaluation of intracranial vessels [3–8]. Advantages include higher vascular contrast and the improved detection and classification of collaterals in ischaemic stroke [9].

With the development of 256- and 320-row CT scanners with 16 cm coverage, whole-brain imaging in a single rotation becomes possible and provides whole-brain 4D-CTA, precluding the need for a separate head CTA. However, since a neck CTA is needed to evaluate the cervical arteries for steno-occlusive disease, most institutes will still perform a separate conventional head and neck CTA acquisition in addition to cerebral CTP. This requires a second injection of contrast material, additional examination time and may result in additional radiation exposure if the scan range overlaps with the cerebral CTP.

We present a novel scanning technique for wide-detector CT scanners that could obviate the need for a separate head and neck CTA acquisition, which we therefore consider a One-Step Stroke Protocol. This technique relies on a wide-detector coverage and a rapid table movement to interleave a whole-brain CTP with neck CTA in one sequence using a single dose of contrast agent. The sequence begins with CTP acquisitions of the brain. At a suitable time point, a neck volumetric CTA (vCTA) is acquired by rapidly moving the table to the adjacent neck region. Then, the table moves back to the brain, where the CTP acquisition is continued. By using a single exam, imaging time, radiation exposure and the amount of contrast material can be reduced.

This article provides the proof-of-concept of this technique and assesses the image quality of the resulting neck CTA acquisition.

## Materials and methods

### Patient group

Approval for this prospective study was obtained and informed consent was waived by the ethics committee of our institution, as in our institution a cerebral NCCT, cerebral CTP, and head and neck CTA is routinely performed in the workup of acute ischaemic stroke patients. One or two time points of the CTP acquisition was substituted for a volumetric neck CTA, leaving all other factors unchanged. In a previous retrospective study (part I), we showed that the calculated perfusion values were not significantly affected.

Four senior technicians were trained to use the One-Step Stroke Protocol on our scanner. Consecutive patients with the indication of acute ischaemic stroke were prospectively enrolled and underwent NCCT, the One-Step Stroke Protocol, and conventional head and neck CTA between November 2013 and June 2014. Inclusion criteria were as follows: admission within a 9-h time window from the onset of their symptoms, NIHSS score of at least 2, and patients presenting during office hours while one of the four trained technicians was available for the exam. Exclusion criteria were as follows: patients who did not receive conventional CTA, severe metal artefacts on conventional CTA or the One-Step Stroke Protocol, severe motion artefacts resulting from motion during scanning on conventional CTA or the One-Step Stroke Protocol, known kidney failure and previous allergic reactions to iodinated contrast medium.

### Imaging protocol

CT imaging was performed on a 320-detector row CT scanner (Aquilion ONE; Toshiba Medical Systems, Otawara, Japan). The scan protocol consisted of a cerebral NCCT, the One-Step Stroke Protocol, and conventional head and neck CTA. In all patients two contrast injections were performed, one for the One-Step Stroke Protocol and one for CTA.

NCCT scanning of the brain was performed at 120 kV tube voltage and 280 mA tube current, 1 s rotation time, 0.5 mm section thickness and 0.5 mm reconstruction interval.

**Fig. 1:**
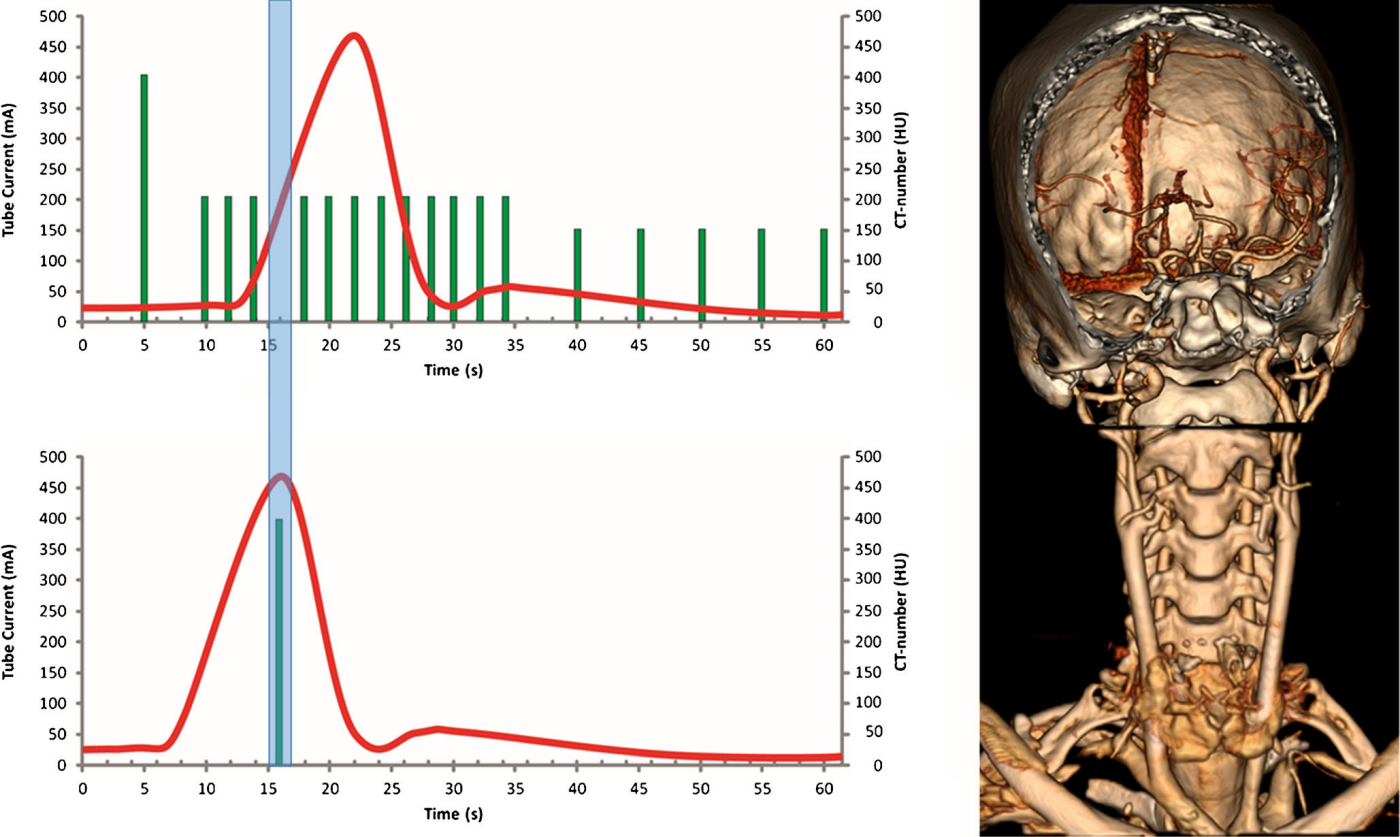
Schematic overview of the One-Step Stroke Protocol with 3D volume rendering of the head CTP and volume neck CTA. The One-Step Stroke Protocol consists of a 16-cm volumetric whole-brain CTP acquisition that is interrupted as soon as contrast material is detected in the arteries of the central slab of the 3D volume. Within 1.8 s, the table is then rapidly moved to the neck, where a 16-cm volumetric scan is performed with 0.5 s acquisition time. The table is then automatically moved back to the brain to resume the CTP acquisition. Note that the arterial enhancement in the neck is excellent because the contrast reaches the neck earlier than the brain and the enhancement curve in the neck is shifted to the left

For the One-Step Stroke Protocol, 50 ml non-ionic contrast agent (300 mg iodine/ml Xenetix 300; Guerbet, Villepinte, France) was injected into an antecubital vein with an injection rate of 5 ml/s, followed by a 40-ml saline flush at 5 ml/s. Whole-brain volumetric acquisitions (16 cm z-coverage) were acquired with 0.5 mm slice thickness, 0.5 s rotation time and 80 kV tube voltage. The CTP acquisition started 5 s after contrast injection, with 1 volumetric acquisition at 200 mAs, followed after 4 s by 12 volumetric acquisitions each at 100 mAs with 2 s sampling interval, followed after 5 s by five acquisitions each at 75 mAs with 5 s interval. The total number of volumetric acquisitions was 18 and the total scan duration was 60 s (Fig. 1). Image reconstruction was done with reconstruction kernel FC41 and standard AIDR3D (adaptive iterative dose reduction in three dimensions; Toshiba Medical Systems).

During the CTP acquisition the table was moved to the neck and back to the brain to perform a neck CTA. This movement required 2 × 1.8 s with our CT scanner. The neck CTA can be acquired in 0.275–0.500 s. Therefore, the time gap induced between adjacent CTP acquisitions can theoretically be less than 4 s. At present, the neck CTA is initiated manually as soon as arterial contrast enhancement is visible on the mid-section of the brain, which is presented as a real-time reconstruction on the scanner console. This manual interaction increases the time gap slightly. Given a scanning sequence in which data are acquired every 2 s, the time gap needed to acquire the neck CTA is in the range of one to two volumetric acquisitions of the CTP (Fig. 1).

Since bolus tracking during CTP is not available yet, a manual interaction of the technician was needed to start the neck CTA as soon as contrast material was visible in the central slice of the brain during the CTP acquisition. The table then moved to the neck within 1.8 s. The volumetric neck CTA (with 16 cm z-coverage) was acquired with following parameters: 0.5 mm slice thickness, 0.5 s rotation time, 80 kV tube voltage and 200 mAs exposure. FC43 filter was used for image reconstruction. After acquiring the neck CTA, the table moved back to the brain to resume the CTP acquisition.

For the head and neck CTA, 70-80 ml non-ionic contrast agent (300 mg iodine/ml Xenetix 300; Guerbet, Villepinte, France) was injected into the antecubital vein with an injection rate of 5 ml/s followed by a 40-ml saline flush with an injection rate of 5 ml/s. The CTA covered the area from just below the aortic arch to the vertex. CTA was performed in a helical scan mode using the following parameters: 80 × 0.5 collimation, 0.81 pitch, 120 kV, automatic exposure control with standard deviation of 10 and exposure range 100-700 mA, 0.5 mm and 3.0 mm slice thickness, 0.5 s rotation time, reconstruction filter FC43 and standard AIDR3D. The bolus tracker was set at an absolute threshold of 180 HU at the level of the descending aorta.

### Feasibility

The distance from the lowest origin of the common carotids to the vertex was measured on conventional CTA to determine the ideal total z-coverage. Given a margin of 1 cm for the coverage of the One-Step Stroke Protocol, we determined how many patients could have had complete coverage of the craniocervical circulation covered under ideal planning of the scan range. For this we determined the number of patients in whom this distance was less than 31 cm. The distance missed of the origin of the left common carotid artery by the One-Step Stroke Protocol was recorded in centimetres. Also, the heights of the patients were reported.

In addition, we measured the time interval between the last CTP acquisition, before performing the volumetric neck CTA, to the first CTP acquisition thereafter.

### Quantitative analysis

Arterial enhancement of the carotid and vertebral arteries was measured at three levels: just above the origin of the common carotid artery (CCA), in the internal carotid artery (ICA) above the bifurcation and at the level of the C1-C2. Enhancement of the vertebral arteries was measured at the same three levels. For contrast-to-noise ratio (CNR) the sternocleidomastoid muscle and the noise represented by the standard deviation of the HU values in a region of interest (ROI) in the surrounding air was used. The ROIs were kept constant at 4 mm^2^ and 64 mm^2^ for the arterial enhancement and sternocleidomastoid muscle, respectively. Calcifications and plaques were avoided.

### Qualitative analysis

Three observers (F.J.A.M., E.J.S. and M.P. with 10, 5 and 20 years of experience in stroke imaging, respectively) qualitatively scored image quality at several levels of the neck CTA acquisitions. Data was anonymised and observers were blinded to the technique. This was realised by cropping both images of the conventional CTA and the One-Step Stroke Protocol neck CTA such that only the overlapping field of view of both techniques was visible and that the z-coverage could not reveal the used scanning technique. Image quality was scored on a scientific scoring workstation Cirrus, developed at the Diagnostic Image Analysis Group (DIAG), Nijmegen, The Netherlands.

Image quality of the carotid and vertebral artery was determined on the following five-point scale: 1, non-diagnostic; 2, poor image quality, sufficient for vascular evaluation; 3, moderate image quality, sufficient for soft tissue and vascular evaluation; 4, good image quality; 5, excellent image quality [10].

Streak and pulsation artefacts were scored as follows: 1, severe artefacts, non-diagnostic quality; 2, substantial artefacts, moderate impairment of diagnostic quality; 3, clearly visible artefacts, no impairment of diagnostic quality; 4, hardly visible artefacts; 5, no artefacts.

Artefacts and image quality of cervical arteries were scored at the same three anatomic levels that were used for quantitative evaluation of the arterial enhancement.

### Diagnostic accuracy

An expert observer (F.J.A.M.) assessed vCTA and conventional CTA for the presence of stenoses, occlusions, dissections, and coverage. Stenoses were graded as significant (more than 50 % luminal stenosis) or non-significant (less than 50 % luminal stenosis). The observer was blinded to technique, clinical information and diagnoses. The images were shown in a random order. Multiplanar reconstructions (MPRs) were available. Scans were presented with equal display settings, but the observer could change the settings according to clinical practice (window levelling, arbitrary planes, slab thickness).

### Statistical analysis

Statistical analyses were performed using the Statistical Package of Social Sciences version 20.0 for Windows (SPSS, Chicago, USA). Wilcoxon signed rank test was used to test for significant differences between the image quality of the conventional CTA and the volumetric CTA from the One-Step Stroke Protocol. A *P* value of <0.05 was considered significant.

## Results

### Study population

A total of 25 patients were scanned with the One-Step Stroke Protocol. In three patients, conventional CTA was not performed because of renal insufficiency. In two patients, severe motion artefacts were present on the conventional CTA of the neck. The remaining 20 patients (median age, 72.5 years; age range, 22–90 years; 9 women, 11 men) were included in this evaluation. Patients’ charts were reviewed and the final diagnoses after clinical follow-up of our study population were as follows: acute ischaemic stroke (*n* = 16), transient ischaemic attack (*n* = 1), ischaemic stroke due to vasospasm after subarachnoid haemorrhage (*n* = 1), epileptic seizure (*n* = 1) and slow progressive aphasia (*n* = 1).

### Feasibility

All 20 patients that were enrolled in this study had a successful examination using the One-Step Stroke Protocol. The average distance from the left common carotid origin to the vertex was 312 mm (range, 272-351 mm). In 10 out of 20 patients this distance was smaller than 31 cm. The mean distance missed at the origin of the left common carotid artery was 1.6 ± 0.8 cm (range, 0.5–3.5 cm). The mean height of our 17 patients was 1.8 ± 0.09 m (three patients lacked height data).

The average time gap between adjacent CTP acquisitions for the interleaved neck CTA was 5.9 ± 0.3 s (range, 5.4-6.5 s).

### Quantitative analysis

Mean arterial enhancement and CNR are reported in Table 1. The arterial enhancement was significantly higher for the vCTA of the One-Step Stroke Protocol than for the conventional CTA. Near the origin of the CCA, average HU values were 477 ± 148 on vCTA versus 396 ± 83 at CTA (*P* = 0.004). In the ICA near the bifurcation, average HU values were 579 ± 118 on vCTA versus 425 ± 87.4 on CTA (*P* < 0.001). In the distal ICA near the skull base, we measured 512 ± 116 HU on vCTA versus 441 ± 100 HU on CTA (*P* = 0.006). HU values in the vertebral arteries were significantly higher at the level above the carotid bifurcation and at the skull base but not at the level of the origin of the CCA.

**Table 1:** Attenuation numbers and contrast-to-noise ratios (mean ± standard deviation)

		CTA		vCTA		*P* value	
Level	Artery	HU	CNR	HU	CNR	HU	CNR
Origin	CCA	396 ± 83	39 ± 12	477 ± 148	46 ± 30	0.004*	0.183
	Vertebral arteries	331 ± 81	31 ± 12	354 ± 111	31 ± 19	0.264	0.965
Bifurcation	ICA	425 ± 87	42 ± 12	579 ± 118	57 ± 31	0.000*	0.014*
	Vertebral arteries	392 ± 91	38 ± 13	464 ± 98	43 ± 23	0.001*	0.215
C1-C2	ICA	441 ± 100	44 ± 14	512 ± 116	50 ± 28	0.006*	0.302
	Vertebral arteries	398 ± 81	39 ± 11	453 ± 110	41 ± 22	0.037*	0.602

*P ˂ 0.05, significant

*CNR* contrast-to-noise ratio, *CCA* common carotid artery, *ICA* internal carotid artery, *vCTA* volumetric CTA as part of the One-Step Stroke Protocol

Except for the ICA near the level of the carotid bifurcation, the CNR in the cervical arteries did not differ significantly, although average HU values for vCTA were higher than for CTA.

### Qualitative analysis

The subjective image quality scores are summarised in Table 2. Mean subjective image quality scores in the ICA at the level of the bifurcation and at the level of the C1-C2 did not significantly differ between the two techniques. However, at the level of the origins of the CCA two of the three observers rated the mean subjective image quality lower for vCTA than for conventional CTA.

**Table 2:** Subjective image quality scores

		Reader 1			Reader 2			Reader 3		
Level	Criterion	CTA	vCTA	*P* value	CTA	vCTA	*P* value	CTA	vCTA	*P* value
Origin	Quality CCA^a^	3.0 ± 0.7	2.4 ± 0.7	0.003*	3.9 ± 0.4	3.8 ± 0.4	0.414	3.8 ± 0.4	3.3 ± 0.7	0.026*
	Quality VA^a^	3.0 ± 0.7	2.4 ± 0.7	0.006*	3.9 ± 0.3	3.5 ± 0.7	0.013*	3.8 ± 0.4	3.3 ± 0.7	0.008*
	Streak artefacts^b^	2.7 ± 0.6	2.5 ± 0.7	0.001*	1.0 ± 0.2	1.7 ± 0.6	0.000*	1.5 ± 0.7	2.2 ± 0.6	0.001*
	Pulsation artefacts^b^	2.7 ± 0.6	2.3 ± 0.7	0.013*	1.1 ± 0.4	1.1 ± 0.3	0.655	1.5 ± 0.7	1.3 ± 0.7	0.160
Bifurcation	Quality ICA^a^	3.7 ± 0.5	3.3 ± 0.7	0.109	4.0 ± 0.0	4.0 ± 0.0	1.00	4.0 ± 0.0	4.0 ± 0.0	1.00
	Quality VA^a^	3.7 ± 0.5	3.3 ± 0.7	0.109	4.0 ± 0.0	4.0 ± 0.0	1.00	4.0 ± 0.0	4.0 ± 0.0	1.00
	Streak artefacts^b^	1.2 ± 0.4	1.4 ± 0.6	0.727	1.0 ± 0.0	1.0 ± 0.0	1.00	1.0 ± 0.2	1.2 ± 0.4	0.180
	Pulsation artefacts^b^	1.1 ± 0.3	1.3 ± 0.6	0.453	1.0 ± 0.0	1.0 ± 0.0	1.00	1.0 ± 0.0	1.0 ± 0.0	1.00
C1-C2	Quality ICA^a^	3.3 ± 0.6	3.4 ± 0.8	0.754	4.0 ± 0.0	4.0 ± 0.2	0.317	4.0 ± 0.2	4.0 ± 0.0	0.317
	Quality VA^a^	3.3 ± 0.6	3.4 ± 0.8	0.754	4.0 ± 0.2	4.0 ± 0.0	0.317	4.0 ± 0.2	4.0 ± 0.0	0.317
	Streak artefacts^b^	1.7 ± 0.6	1.6 ± 0.5	1.000	1.2 ± 0.4	1.2 ± 0.4	1.00	1.6 ± 0.6	1.7 ± 0.5	0.414
	Pulsation artefacts^b^	1.0 ± 0.2	1.1 ± 0.5	1.000	1.0 ± 0.0	1.0 ± 0.0	1.00	1.0 ± 0.0	1.0 ± 0.0	1.000

* *P* ˂ 0.05, significant

^a^Higher numbers indicate better image quality

^b^Lower numbers indicate fewer artefacts

Artefacts were not rated as severe, hampering diagnostic evaluation (score 1) in any of the exams. There was no significant difference in artefact scores in the bifurcation region and the skull base region between vCTA and CTA. Near the origins of the CCA, artefact scores were significantly worse for vCTA (Table 2). One observer rated pulsation artefacts at the level of the origins of the CCA significantly worse on vCTA than on CTA. All other artefact scores did not significantly differ between the two techniques.

**Fig. 2:**
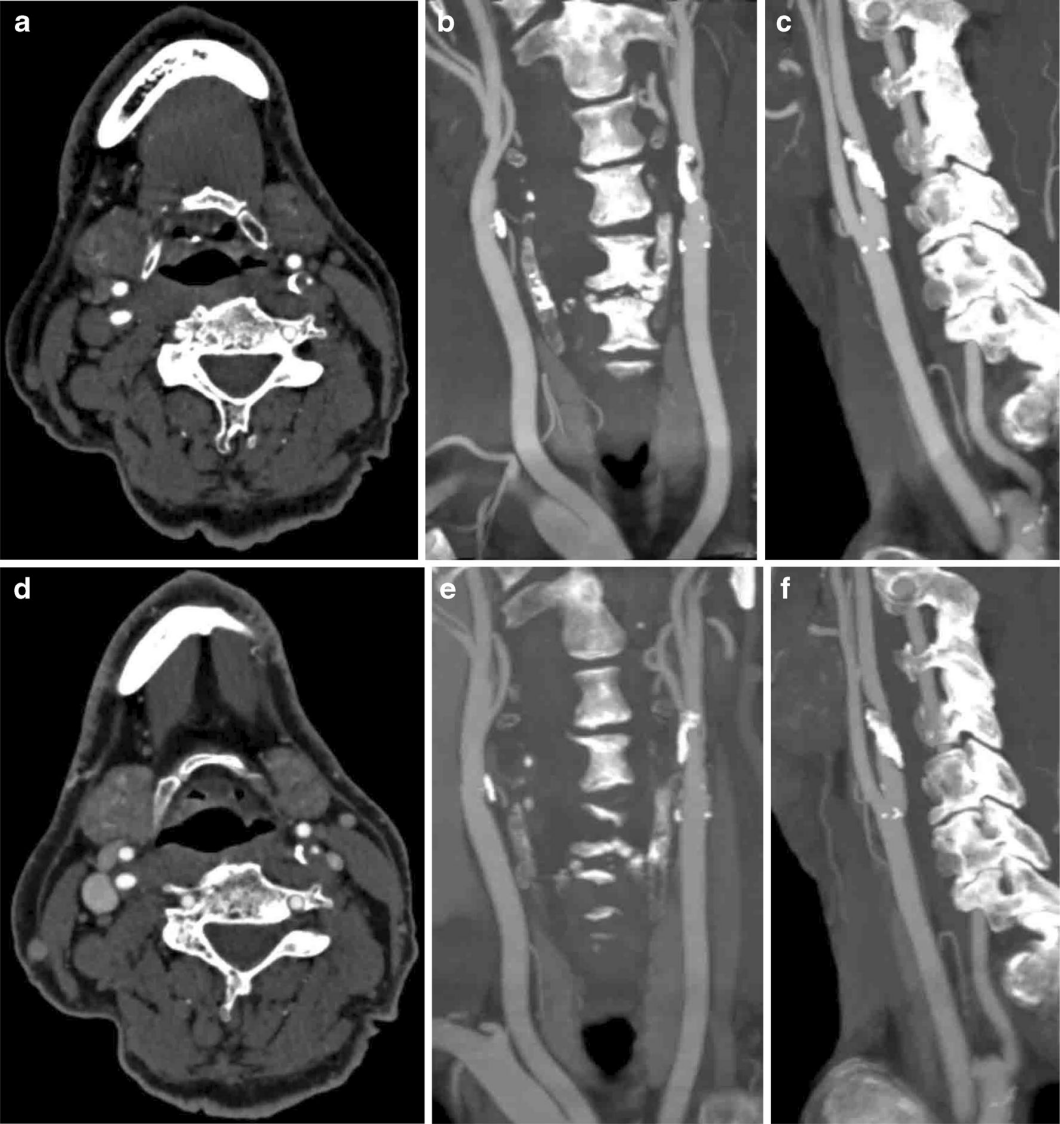
Comparison of the neck CTA of the One-Step Stroke Protocol (**a**, **b** and **c**) and the conventional CTA (**d**, **e** and **f**) in a patient with sudden weakness of the right hand and leg. **a** and **d** Axial images at the level above the carotid bifurcation (window centre, 200 HU; window width 700 HU). The left internal carotid artery shows a pinpoint stenoses, plaque and calcifications. In this subject, the origin of the left common carotid artery was missed by 2 cm. Image quality was rated equally good at the level of the carotid bifurcation between the One-Step Stroke Protocol and the conventional CTA protocol. **b** and **e** Coronal maximum intensity projections with 10 mm reconstructions; **c** and **f** are sagittal maximum intensity projections with 10 mm reconstructions (window centre, 200 HU; window width 700 HU)

Comparison of neck CTA of the One-Step Stroke Protocol and conventional CTA is illustrated in Fig. 2.

### Diagnostic accuracy

Full agreement was noted regarding the presence of stenoses, occlusions or dissection between the conventional CTA and vCTA. However, in one case, a carotid bifurcation stenosis was graded as non-significant on conventional CTA, while no stenosis was mentioned on vCTA; after reviewing the images next to each other, this was rather due to observer variability than to the imaging technique. No stenoses were found at the origin of the left common carotid artery, brachiocephalic artery or vertebral arteries in the conventional CTA; therefore, no stenoses were missed due to lack of coverage of vCTA in our study population. Results are shown in Supplementary Table 3.

## Discussion

Our study shows that it is feasible to interleave cerebral CTP with volumetric neck CTA in a single One-Step Stroke Protocol. Neck CTA derived from a One-Step Stroke Protocol provides better arterial enhancement and similar image quality in the carotid bifurcation region and the skull base compared with conventional CTA, but suffers from artefacts in the lower neck.

Removing a conventional CTA acquisition from the CT stroke workup reduces the total amount of contrast by eliminating a second contrast injection. In addition, the overall scanning time can be reduced. Radiation exposure is lower compared with a brain CTP combined with a separate conventional head and neck CTA acquisition. The dose-length product (DLP) for the proposed One-Step Stroke Protocol was 2,262 ± 144 mGy·cm and 2,447 ± 241 mGy·cm for the conventional cerebral CTP and separate head and neck CTA.

A separate cerebral CTA appears not to be necessary: the quality of vascular information derived from CTP data is sufficient to omit covering the brain during a separate CTA. In fact, grading of collateral vessels using 4D-CT has been shown to be superior to collateral assessment using conventional CTA [9] for the prediction of outcome of acute ischaemic stroke. Diagnostic performance of a single timing invariant CTA derived from 4D-CTA has been shown to be similar to that of a conventional CTA [7, 9, 11].

**Fig. 3:**
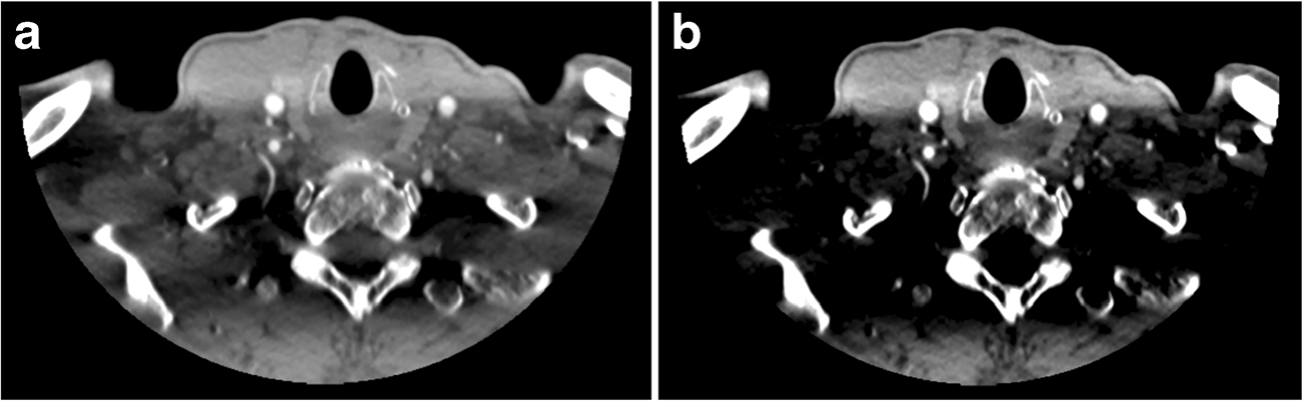
Example of streak artefacts in the lower neck. Using a wide window setting (**a**) of W/L = 900/100, the image quality remains diagnostic for the vascular structures but at a narrower window setting (**b**) of 500/150, these band-like artefacts render some soft tissue structures invisible

Proper timing of contrast material administration is important for good arterial enhancement and image quality of neck CTA. Since the volumetric neck CTA of the One-Step Stroke Protocol is timed immediately after contrast arrival in the cerebral arteries, scanning is performed in the upslope of the arterial enhancement. This is reflected by mean arterial HU values with our technique that are comparable with other studies [12, 13] or even higher [13–15]. The use of lower kV settings also results in higher attenuation in the carotids using the 80 kV of the vCTA compared to the 120 kV of conventional CTA. This is in concordance with several studies with lower kV settings [10, 16]. A side effect is an increase in streak artefacts in the lower neck [16]. Phantom studies showed that the streak artefacts were explained by photon starvation, by the non-utilised contrast agent in the brachiocephalic vein (Fig. 3), and by beam hardening caused by the lower neck [16, 17]. The phantom study also showed that the use of a wide beam width (0.5 × 320) creates scattering of the bone resulting in streak artefacts. The use of smaller beam width will reduce the scattering and therefore the streak artefacts, but will also result in lower coverage which is not desirable [17]. Our study confirms the presence of significantly more streak artefacts in vCTA compared to CTA. While this affected image homogeneity, the artefacts mainly affect soft tissue and not the vessels. Therefore image quality was rated sufficient for diagnostic evaluation of the arteries in this region. Also, the diagnostic accuracy study showed full agreement for both CTA techniques. Further optimisation of image reconstruction from volumetric acquisitions can be expected to alleviate this problem. Image quality in the rest of the neck was rated good to excellent, and was similar to that of conventional CTA.

The new protocol has limitations. First, omitting one or two a time points from the CTP acquisition might affect the accuracy of CTP values. In part I, we demonstrated that a 4- to 6-s time gap in the CTP acquisition has only minor effects on CTP values, which is in line with another study [18]. Skipping three time points would result in an 8-s time gap, which is unfavourable for the evaluation of CTP tissue attenuation curves [18] and should therefore be avoided as much as possible. In theory, a time gap of 4 s would suffice for the interleaved volumetric neck CTA. However, due to technician reaction time and the reconstruction time of the central slice, the measured interval was 5.9 ± 0.3 s, up to 6.5 s time gap. The cases with up to 6.5 s time gap concerned mainly the first cases scanned with the One-Step Stroke Protocol and can to some extent be attributed to the learning curve of the technicians. Bolus tracking on CTP with automated timing of the neck CTA acquisition would probably be of added value for reducing the demand on the technician and to keep the resulting time gap as short as possible.

Second, a potential limitation of the technique is the total coverage of the combined neck and cerebral CTA, which is approximately 2 × 16 cm. Our results show that this range is sufficient in the majority patients to cover the region from the origins of the common carotid arteries to the cerebral vertex (Fig. 1). However, in very tall patients this will not be sufficient. The scout view enables precise planning of the scan range in order to not waste scan length at the vertex and take full advantage of the available coverage. Despite missing the origin of the left common carotid artery and brachiocephalic artery on vCTA, no stenoses were missed at the origin on the conventional CTA in our study population. Note that in all patients the carotid bifurcation was covered on the vCTA, in which (atherosclerotic) steno-occlusive disease is most prevalent and this course is clinically of most importance. Also, the origins of the vertebral arteries were covered in all patients with vCTA. As our study population is relatively small, this limits drawing firm conclusions.

In conclusion, our proof-of-concept study shows that it is feasible to interleave cerebral CTP with neck CTA. The neck CTA provides better arterial enhancement and similar image quality in the region of the carotid bifurcation compared with conventional CTA. Image quality in the lower neck is diagnostic but suffers from streak artefacts. Precise planning of scan range is necessary to cover the left common carotid origin. Under these conditions, CTA obtained from a One-Step Stroke Protocol can be an alternative for the conventional CT scanning protocol, especially when a subsequent head and neck CTA with additional injection of contrast material and radiation exposure is undesirable (e.g. subjects with kidney insufficiency and young patients). Further studies are necessary to validate the technique and to assess diagnostic accuracy in a larger study population.

## Electronic supplementary material

Below is the link to the electronic supplementary material.

ESM 1 (DOCX 38 kb)
