# Supplementary material for: Interleaving cerebral CT perfusion with neck CT angiography. Part II: clinical implementation and image quality
Source: Eur Radiol. 2016 Sep 21;27(6):2411–8. doi: 10.1007/s00330-016-4592-z (PMC5408041; doi:10.1007/s00330-016-4592-z)
Supplement: Supplementary file 1 — (DOCX 38 kb) [file 330_2016_4592_MOESM1_ESM.docx]

## Supplementary Table 3 Diagnostic Accuracy

|  | | CTA | | | vCTA | | |  |
| --- | --- | --- | --- | --- | --- | --- | --- | --- |
| Level | Vascular segment | Non-Significant Stenoses (<50%) | Significant Stenoses (>50%) | Occlusions | Non-Significant Stenoses (<50%) | Significant Stenoses (>50%) | Occlusions | Number of Discrepancies |
|  |  |  |  |  |  |  |  |  |
| Origin | L-CCA | 0 | 0 | 0 | 0 ° | 0° | 0° | 0° |
|  | R-CCA | 0 | 0 | 0 | 0° | 0 ° | 0° | 0° |
|  | L-VA | 0 | 0 | 0 | 0 | 0 | 0 | 0 |
|  | R-VA | 0 | 0 | 0 | 0 | 0 | 0 | 0 |
|  |  |  |  |  |  |  |  |  |
| Bifurcation | L-ICA | 7 | 2 | 0 | 6 | 2 | 0 | 1^*^ |
|  | R-ICA | 3 | 2 | 1 | 3 | 2 | 1 | 0 |
|  | L-VA | 0 | 0 | 1 | 0 | 0 | 1 | 0 |
|  | R-VA | 0 | 0 | 0 | 0 | 0 | 0 | 0 |
|  |  |  |  |  |  |  |  |  |
| C1-C2 | L-ICA | 0 | 0 | 0 | 0 | 0 | 0 | 0 |
|  | R-ICA | 0 | 0 | 1 | 0 | 0 | 1 | 0 |
|  | L-VA | 0 | 0 | 1 | 0 | 0 | 1 | 0 |
|  | R-VA | 0 | 0 | 0 | 0 | 0 | 0 | 0 |

Note - L-CCA = Left common carotid artery, R-CCA = Right common carotid artery, L-VA = Left vertebral artery, R-VA = Right vertebral artery, L-ICA = Left internal carotid artery, R-ICA = Right internal carotid artery.

*In one case, a carotid bifurcation stenosis was graded as non- significant (<50%) on conventional CTA, while no stenosis was mentioned on vCTA; after reviewing the images next to each other this was rather due to observer variability than to the imaging technique.

°In 10 of 20 patients the origin of the left and right common artery was not covered in vCTA and could therefore not be rated.
